# Feasibility and Acceptability of Remote Physical Exercise Programs to Prevent Mobility Loss in Pre-Disabled Older Adults during Isolation Periods Such as the COVID-19 Pandemic

**DOI:** 10.1007/s12603-021-1688-1

**Published:** 2021-10-11

**Authors:** F. Buckinx, M. Aubertin-Leheudre, R. Daoust, S. Hegg, D. Martel, M. Martel-Thibault, Marie-Josée Sirois

**Affiliations:** 1 Centre de recherche, Institut universitaire de gériatrie de Montréal (IUGM), CIUSSS du Centre-Sud-de-l’Île-de-Montréal, Montreal, Canada; 2 Département des Sciences de l’activité physique, Faculté des sciences, Université du Québec à Montréal, Montréal, Canada; 3 Département de médecine de famille et médecine d’urgence, Université de Montréal, Montréal, Canada; 4 Centre d’étude en médecine d’urgence, Hôpital Sacré-Cœur de Montréal, CIUSSS NIM, Montréal, Canada; 5 Centre d’Excellence sur le Vieillissement de Québec, Québec, Canada; 6 Centre de recherche du CHU de Québec-Université Laval, Québec, Canada; 7 Département de réadaptation, Université Laval, Québec, Canada; 8 1435 de Longueuil, Québec, Qc G1S 2G2 Canada

**Keywords:** Remote exercise program, validation, adherence, satisfaction, difficulty, web technology

## Abstract

This study aimed to assess the feasibility and acceptability of remote physical exercise (PE) to prevent mobility loss among pre-disabled older adults during the COVID-19 lockdowns.

Participants followed a 12-week PE remote program in Zoom© supervised groups (Web-Ex group, n=11) or phone-supervised individual booklet-based home-program (Booklet group, n=33).

The total rate of adherence was 82.5% in the Web-Ex group and 85.8% in the Booklet group. The level of satisfaction was « a lot » for 60% of the participants in the Web-ex group and for 37.9% of those included in the Booklet group. Respectively 10% and 31% of the participants rated the difficulty as « low » in the web-ex and Booklet groups.

Remote physical exercise using a web technology or booklets at home with regular and personalized follow-up during the lockdown was feasible and acceptable among pre-disabled seniors.

## Background

**N**ormal aging is often accompanied by a deterioration on functional abilities (1) that can be exacerbated by physical inactivity and sedentary lifestyle, which affect more than 50% of the older adults (2). In addition, this deterioration is associated with restricted mobility among older adults, creating a vicious cycle of deconditioning (2), which accelerates the spiral of loss of physical autonomy and increases the need for health care services (3). Social isolation that hit populations during the COVID-19 lockdowns, has accentuated the problem of inactivity and sedentary lifestyle (4). As such, sedentary time increased from 5 to 8 hours per day during COVID-19’s first wave (5).

Fortunately, previous studies showed that pragmatic web tools integrating physical exercise (PE) programs that are adapted to older adults functional capacities (e.g. SPRINT (6) and MATCH (7) tools), are potential solutions to prevent their physical decline. A recent meta-analysis highlighted that home-based exercises appear effective to improve components of physical-fitness such as muscle strength, muscle endurance, muscle power, and balance (8). Moreover, according to a recent survey, 50% of seniors use the Internet every day and have a tablet, computer or smartphone (9). Thus, implementing remote PE using web technology could be a solution to maintain the health in older adults (10), while avoiding physical contact and risk of contagion. In that sense, Chaabene et al. concluded that, in times of restricted physical activity due to pandemics, home-based exercises are also an alternative to counteract physical inactivity and to keep older adults fit and healthy (8).

While the benefits of PA programs depend on continued participation (i.e. adherence), a change in lifestyle to include regular PE is difficult for many people of all ages. This is especially true in advanced ages (11) as increased co-morbidities, lack of social support, disability or depression in older adults may impede adherence rate to PA programs, which is particularly low in this population (12).

To date, few data related to the feasibility and acceptability of remote PE using web technology among seniors have been published. Thus, we aimed to assess the feasibility and acceptability of this type of intervention among pre-disabled older adults during isolation periods such as the COVID-19 pandemic.

## Methods

### Study design and participants

This is a 12-month intervention study, which started in May 2020 among 44 pre-disabled seniors who were previous participants of the Canadian “Cedecoms” trial (ClinicalTrial. gov: NCT03991598) testing the benefits of physical exercises after visiting the Emergency Department (ED) with a minor injury.

The trial inclusion criteria were: age ≥ 65 years, presenting to ED with a minor injury not requiring hospital admission, independence in daily living activities (eating, toileting and transfers, dressing, showering, walking, continence) in the 4 weeks pre-injury, and discharged home after their ED assessment. Individuals who were hospitalized, living in a long-term care facility, unable to consent to the trial, not speaking English or French were excluded.

The current study is a convenience sample among participants included in the trial in 2019–20.

This research was approved by the REB of the “CHU de Québec-Université Laval” and all participants gave informed consent.

### Intervention

Participants received a 12-week PE remote program (1 hour/3-times/week) in Zoom© supervised groups (Web-Ex group, n=11) or phone-supervised booklet-based individual home-program (booklet group, n=33). PE programs were adapted and specific to pre-disabled seniors, supervised by kinesiologist and each session (zoom© or booklet) started with a 5-min warm-up, followed by 50 min of weight-bearing strengthening, standing balance, and light aerobic exercises, and ended with a 5-min cooldown including stretching.

### Measurements

Participants were evaluated and interviewed by a trained kinesiologist every three months, through zoom© meetings or by phone.

### Feasibility and acceptability of the intervention

Based on the available literature, two indicators of the acceptability were measured (i.e. adherence rate and satisfaction) (13) while a proxy of feasibility was assessed in this study (i.e. the perceived difficulty) (14) : 1) Adherence rate: the proportion of completed week sessions out of the total week sessions prescribed (11). 2) Self-reported satisfaction: every two weeks of PE program, participants were asked to answer a 4-point likert scale (0= not at all, 1= a little, 2= good, 3= a lot) to assess their perceived level of satisfaction with the prescribed program. Then, the average of ratings over the 12-week duration of the program was calculated. 3) Perceived difficulty: every two weeks of PE program, participants were asked to answer a 4-point likert scale (i. 1=low, 2= moderate, 3=difficult, 4= very difficult) to assess their perceived level of difficulty with their program. Then, the average of ratings over the 12-week duration of the program was calculated.

In addition, qualitative interviews were conducted with the participants and the kinesiologists conducting the PE programs, in order to understand the facilitators and barriers to remote PE programs (web technology in groups or individual booklet-based programs). More specifically, participants were asked open-ended questions such as: “what do you think made it easy for you to get involved and to complete your PE program?”, “What made it more difficult?”, “What did you like or dislike about your PE program?” Questions were similar for the kinesiologists, e.g. “What did or did not facilitate conducting such remote PE programs and remote supervision of your participants?”

### Other measures

The systematic review of Picorelli et al. highlighted personal factors associated with adherence to PE program (11). These confounding factors were therefore measured: Demographic factors: age, sex, main occupation, education (years of schooling completed), marital status. Health-related factors: perceived health (12-item Short-Form Health Survey: SF-12). Physical health factors: Body Mass Index, walking speed (timed 4-meter gait speed). Psychological factors: loneliness (UCLA-Loneliness Scale-3), cognitive status (Telephone Interview for Cognitive Status-TICS-m), fear of falling (FES-1 scale).

### Statistical analysis

Data distributions were tested using the Kolmogorov test. Quantitative variables were expressed as means ± standard deviations (SD). Qualitative variables were expressed as percentages. Independant t test were used to compare the means between groups, and Chi square tests were used to compare proportions. All statistical analyses were performed using SPSS 25.0 (Chicago, IL, USA). P-value ≤ 0.05 was considered statistically significant.

The qualitative analyses consisted of reading each participant’s responses in details and deductively categorizing the main ideas that emerged into themes. The small number of people interviewed did not require full transcriptions and coding into software such as QDA miner.

## Results

### Participants

Table 1 presents the baseline characteristics of the 44 participants included in the 12-week intervention. At baseline, both groups were comparable regarding sociodemographic data, excepted for the level of education and the perceived general health which were higher in the Web-Ex group compared to the Booklet group (p=0.02 and p=0.01, respectively).

**Table 1:** Participants’ baseline characteristics (n=44)

	**Total participants (n=44)**	**Web-Ex group (n=11)**	**Booklet group (n=33)**	**p-value**
Socio-demographic factors				
Sex (women)	30 (68.1)	8 (72.7)	22 (71)	0.91
Age (years)	79.3 ± 6.2	77 ± 6.9	80.1 ± 5.9	0.41
Main occupation				0.55
- Full- or part- time work	1 (2.3)	0 (0)	1 (3.03)	
- Retirement	41 (93.2)	10 (90.9)	31 (93.4)	
- Volunteer work	3 (6.8)	1 (3.03)	2 (18.2)	
Education				0.02
- Primary school	5 (11.3)	0 (0)	5 (16.1)	
- High school	8 (18.2)	2 (18.2)	6 (19.4)	
- College	14 (31.8)	3 (27.3)	11 (35.4)	
- University	15 (34.1)	6 (54.6)	9 (29)	
Marital status				0.20
- Married	18 (40.9)	5 (45.4)	13 (41.9)	
- Living with partner	2 (4.5)	1 (9.09)	1 (3.23)	
- Divorced/separated	6 (13.6)	0 (0)	6 (19.4)	
- Widowed	11 (25)	2 (18.2)	9 (23.0)	
- Single, never married	5 (11.4)	3 (27.3)	2 (6.45)	
Health related status				
Perceived general health: SF-12				0.01
- Excellent	1 (2.2)	1 (9.2)	0 (0)	
- Very good	24 (54.5)	3 (29.9)	21 (61.8)	
- Good	12 (27.3)	4 (36.8)	8 (23.5)	
- Fair	6 (13.6)	2 (18.4)	4(11.8)	
- Poor	0 (0)	0 (0)	0 (0)	
Physical factors				
BMI (kg/m^2^)	27.5 ± 5.0	25.8 ± 1.8	28.0 ± 6.1	0.54
Physical function: 4m- gait speed (m/s)	5.5 ± 1.6	5.9 ± 1.9	5.5 ± 1.9	0.79
Psychological factors				
Loneliness : UCLA score (/9)	4.3 ± 1.6	3.8 ± 1.3	4.3 ± 1.4	0.11
Cognitive status : TICS (/50)	36.9 ± 9.1	34. 9 ± 12.7	34.8 ± 10.1	0.31
Fear of falling : FES-I (/28)	4.4 ± 4.1	3.6 ± 4.4	4.4 ± 4.1	0.06

Legend : SF-12: 12-item Short Form Survey; BMI:Body Mass Index ; TICS: Telephone Interview for Cognitive Status; FES-I : Falls Efficacy Scale International

### Feasibility and acceptability of the intervention

#### Adherence rate

The total adherence rate (proportion of completed week sessions out of the total week sessions prescribed) was 82.5% (9.9 ± 3.4/12 weeks) in the Web-Ex group and 85.8% (10.3 ± 3.2/12 weeks) in the Booklet group.

**Figure 1:**
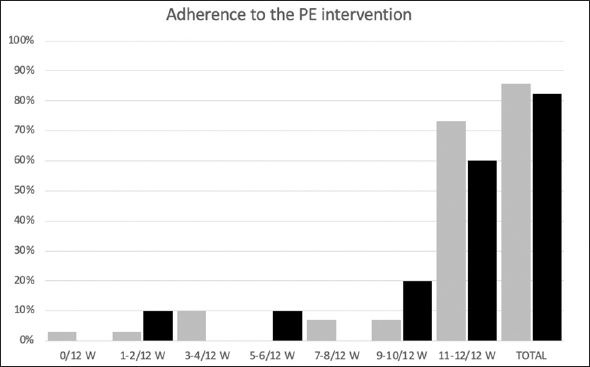
Adherence rate to the intervention, by group Legend: W= weeks

More specifically, in the Web-Ex group, 10% of the participants followed 2/12-week of the program, 10% followed 5–6/12 weeks, 20% followed 9–10/12 weeks and 60% followed 11–12/12 weeks. In the Booklet group, 3.3% of the participants followed 1/12-week of the program, 10% followed 3–4/12 weeks, 6.7% followed 7–8/12 weeks, 6.7% followed 9–10/12 weeks and 73.3% followed 11–12/12 weeks (Figure 1).

During the 12-week intervention, 7 participants dropped out: 5 in the Booklet group (16%, including 1 due to COVID-19 positive) and 2 in the WEB-Ex group (18%).

#### Self-reported satisfaction

**Figure 2:**
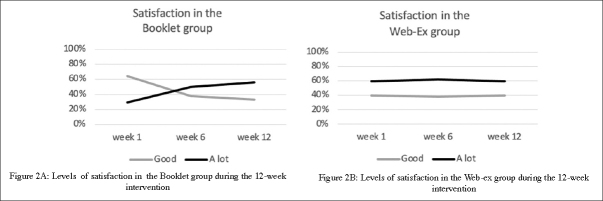
Levels of satisfaction in participants during the 12-week intervention, according to groups (Figure 2A : Booklet group; Figure 2B: Web-Ex group

On average (mean on 12-week PA program), 4 participants (40%) in the Web-Ex group expressed « good » satisfaction with their PE program, while 7 (60%) were « a lot» satisfied. In the Booklet group, the level of the satisfaction was « a little» (1 participant (3.5%)), « good » (19 participants (58.6%)) and « a lot » for 13 of them (37.9%). In addition, as shown in Figure 2, the level of satisfaction remained constant throughout the intervention period in the Web-Ex group while it has improved in the Booklet group in which the proportion of subjects who were satisfied «a lot» with the PE program increased between the first (W1) and the 12th week (W12). Conversely, the proportion of subjects with «good» satisfaction decrease between W1 and W12.

#### Perceived difficulty

In the Web-Ex group, 10 participants estimated the overall difficulty of their PE program with 9 of them (90%) rating it as « moderate » and 1 (10%) rating it as « low ». Interestingly, at W1, 4 participants (40%) rated the difficulty as « low » and 6 (60%) as « moderate », but at W12, 3 participants (33.3%) rated the difficulty as « low », 6 (55.6%) as « moderate » and 1 (11.1%) as « difficult ».

In the Booklet group, 29 participants estimated the difficulty of the PE program. Among them, 20 (69%) rated the difficulty as « moderate », while 9 (31%) rated it as « low ». Similarly to the other group, at W1, 10 participants (36%) of the Booklet group rated the difficulty as « low », 17 (57%) as « moderate » and 2 (7%) as « difficult » but at W12, 8 participants (29%) rated the difficulty as « low » and 21 (71%) as « moderate ».

#### Barriers and facilitators

The participants from the Booklet group identified the following facilitators to their remote PE program: the flexible schedule to perform physical activity sessions, the individual online follow-up and the continuity of services from the kinesiologists throughout the intervention. The participants from the Web-Ex group noted the following facilitators to their remote PE program: the motivation generated by group training, and by the novelty (i.e. new experience), the regular help and follow-up from the kinesiologists, and the fixed and prepredetermined schedule.

From the investigators point of view, the facilitators to remote PE using web technology were: no need to travel, the motivation to try something new, the possibility of maintaining interactions and exercigesing with patients despite the COVID-19 lockdown, remaining available and accessible between sessions for questions or problem solving, sending programs (Booklet group) according to participants’ preferred mean (at the door step, email or regular mail) and real time adaptation and feedback for Web-ex participants. In addition, kinesiologists reported that their constant professional commitment made participants feel they were taking care of and helped maintain their adherence to PE programs.

Participants and kinesiologists reported both barriers to remote PE to be linked to 1) the technology itself: variety of systems (PC, MAC, tablets, computers), difficulty of understanding and using technology devices, need for teaching technology to older adults, and to 2) the clinical aspects: test setup and safety, difficulty to provide remote assistance, to see the whole body during zoom sessions and to provide feedback on exercises as well as difficulty to perceive pain and restrictions or injury (feeling of insecurity).

## Discussion

This study aimed to assess the feasibility (adherence rate and self-reported satisfaction) and acceptability (perceived difficulty) of remote PE among pre-disabled older adults to preserve health during isolation periods such as the COVID-19 pandemic.

A high adherence rate to PE program above 80% in both intervention groups was observed, suggesting that pre-disabled older adults are capable of sustaining PE for 12 weeks. These results are better that the 58 to 77% average adherence rates previously reported among older adults 11 and could potentially be explained by the participants’ socio-demographic characteristics. As indicated by Picorelli et al., higher socioeconomic status and living alone are associated with better adherence to PE program 11 along with presenting fewer health conditions, better self-rated health, better physical and cognitive abilities and fewer depressive symptoms 11. Although our participants were pre-disabled older adults, they shared many favorable characteristics to good adhesion (see Table 1). As mentioned in the qualitative interviews, even if remote, active communication and personalized monitoring by the kinesiologists (health specialist in exercise) contributed to our high adherence rates 15.

With regards to satisfaction with PE programs, there is evidence suggesting that satisfaction is a critical enabling factor for older people to engage in PE 16. With 95% of our participants from both intervention groups expressed at least good satisfaction with their PE programs, it is very likely that satisfaction contributed to the high adherence rate we observed.

Our results also indicate that the perceived level of difficulty was low to moderate in both intervention groups and the adherence rate was high, suggesting that the prescribed PE programs were acceptable. Our findings are in line with previous studies demonstrating that home-based exercise technology is acceptable in various population such as community-dwelling older adults who sustained a minor injury 17, nursing home residents with and without mild cognitive impairments 18 but also among pre-disabled older adults 19.

Besides the professional follow-up mentioned above, another important facilitator to remote PE intervention that emerged from our study, from both investigators and participant’s point of view, is the motivation to try something new. We can hypothesize that the investigators motivated by the study helped to motivate participants to take part and adhere to the intervention. According to a publication understanding motivations to participate in a research study, the role of the investigators is important. Effectively, several participants participate in the study 20. Understanding of motivational factors for participation in and adherence to, exercise programmes is of great importance to older people, health professionals and society as more than 50% of older adults are inactive or sedentary.

Despite our results add to a growing literature focused on investigating remote PE interventions, some potential bias and limitations must be acknowledged. First, a selection bias is possible because of the selection criteria for this study (e.g. volunteer subjects, access to a computer or tablet or smartphone). Because only pre-disabled older adults were included, participants are not representative of the general older adults population. The small sample size also limits the statistical power and the generalizability of our conclusion to larger older adults populations. Then, a social desirability bias is possible since participants knew they were being assessed throughout the study. In addition, we have a potential measurement bias because of the online modality. Effectively, online assessments could affect the reliability and reproducibility of the tests (greater margin of error measurements, viewing angle, lack of postural control, response times from participants). Finally, an information bias is possible since date were self-reported. The judgment of the investigators plays a role in participants’ responses.

## Conclusion

Remote physical exercise using web technology or booklets with regular and personalized follow-ups during the COVID-19 lockdowns seemed feasible and acceptable among pre-disabled seniors. The adherence rate was high in both modalities. However, the level of satisfaction was higher in the webex group while the perceived difficulty was higher in the booklet one. Both interventions web technology or booklet with regular and personalized follow-up could help to prevent deconditioning and loss of autonomy in older adults while allowing social interaction during a lockdown period.
